# How Older Adults Cope with Cognitive Complexity and Environmental Constraints during Dual-Task Walking: The Role of Executive Function Involvement

**DOI:** 10.3390/ijerph16101835

**Published:** 2019-05-23

**Authors:** Roberta Forte, Caterina Pesce, Angela Di Baldassarre, John Shea, Claudia Voelcker-Rehage, Laura Capranica, Giancarlo Condello

**Affiliations:** 1 Department of Human Movement and Sports Sciences, University of Rome Foro Italico, 00135 Rome, Italy; roberta.forte@uniroma4.it (R.F.); laura.capranica@uniroma4.it (L.C.); 2 Department of Medicine and Aging Sciences, “G. d’Annunzio” University of Chieti-Pescara, 66100 Chieti, Italy; a.dibaldassarre@unich.it; 3 Department of Kinesiology, Indiana University, Bloomington, IN 47405, USA; jbshea@indiana.edu; 4 Jacobs Center on Lifelong Learning and Institutional Development, Jacobs University Bremen, 28759 Bremen, Germany; c.voelcker-rehage@jacobs-university.de; 5 Graduate Institute of Sports Training, Institute of Sports Sciences, University of Taipei, Taipei City 111, Taiwan; giancarlo.condello@gmail.com

**Keywords:** cognition, working memory, gait, obstructed walking, physical activity, aging

## Abstract

This cross-sectional study investigated the interactive dual-task (DT) effects of executive function demands and environmental constraints on older adults’ walking and the moderating role of habitual physical activity (PA). Locomotor performance under different environmental constraints (flat versus obstructed walking) and cognitive performance with different executive function involvement (backward counting versus random number generation) were assessed under single-task (ST) and DT conditions in 135 participants (mean age 68.1 ± 8.4). The weekly number of daily steps was measured. Reciprocal DT effects of walking on cognitive performance and of the cognitive task on gait performance were computed and submitted to analyses of covariance with age, PA level, and cognitive functioning as covariates, followed by linear regressions with PA level as predictor. Cognitive task demands and environmental constraints individually and jointly affected gait variability (*p* = 0.033, η_p_^2^ = 0.08) and executive function performance (*p* = 0.009, η_p_^2^ = 0.09). Physical activity level predicted a low but significant percentage of variance of DT effects on gait only in flat walking (R^2^ = 0.04, *p* = 0.027). Results suggest that older individuals may adopt variable task prioritization in dual tasking depending on the type of executive function involvement and the environmental constraints on walking. Their DT ability was slightly affected by habitual PA.

## 1. Introduction

Walking is a major component of physical activity and human mobility, defined as the ability to autonomously move in the environment [1]. Thus, insufficient walking is a main determinant of the 23% of adults, estimated worldwide, who do not meet global recommendations on physical activity for health [2]. Walking has very high ontogenetic relevance, as it is “one of the first things an infant wants to do, and one of the last any of us wants to give up” ([3], cited in Reference [4], p. 6). The onset of the ability to walk has a wide-reaching impact on whole child development [5] and its offset in older adults with chronic conditions is a major cause of a cascade of adverse events compromising health and quality of life [6]. The cost to society of insufficient walking is underestimated when costs outside the health system are considered. For example, the high use of fossil fuel for transportation may be reduced by the increased use of walking as a means of local transportation [2]. Thus, reduced walking may be an important determinant of costs from individual, societal, and environmental perspectives. 

Functional mobility is a multifaceted competence that relies on physical capacities and their interplay with cognitive function [7,8], especially with executive function, which is responsible for goal-oriented actions and behavioral adaptability [9]. This is particularly evident with increasing age, when gait progressively loses automaticity and imposes higher demands on already declining frontal-dependent executive control resources [10,11]. Such parallel losses in motor coordination and executive function challenge the ability to cope efficiently with mobility tasks. 

The decline in gait performance, as reflected in worse and more variable values of spatiotemporal gait parameters are observed particularly when locomotion is performed with a concurrent task (dual-task, DT) [12,13]. How older adults cope with walking in DT conditions with different environmental constraints or cognitive demands is an issue of actual debate, since testing and understanding this complex relationship may help tailor appropriate interventions for fall prevention [14,15]. In fact, aging research has shown a significant association between fall history and walking performance in DT [16]. Dual-task-related falls may be explained by the increased difficulty in prioritizing motor performance over a secondary task [17]. 

According to the “posture first” strategy, older individuals, when faced with DT performance, prioritize the postural task at the expense of the cognitive task to prevent falls [18]. However, integrated models of task prioritization suggest it to be not invariant but dependent on individual characteristics, environmental factors, and instructions, and the complexity of motor and cognitive task demands [17,19,20,21,22]. 

Meta-analyses on the role of cognitive task complexity has not provided definitive conclusions. Some studies found an increasing complexity linked to decrements in gait performance [23], while others showed similar effects for simple and difficult cognitive tasks [24]. This discrepancy is probably due to the methodological heterogeneity of cognitive tasks, which may involve executive function to different degrees. Counterintuitively, a concurrent cognitive task may not always be detrimental, but advantageous for gait performance. Evidence shows that postural control and walking tasks may be performed more efficaciously if the focus of attention is shifted away from those highly practiced tasks [25]. Nevertheless, this benefit disappears in older adults due to resource competition when the demands of the concurrent cognitive task are increased [26]. 

Dual task studies have commonly manipulated either the complexity of the cognitive task or the environmental conditions to alter locomotor demands, whereas the combined effects are still under-investigated [20,27,28]. Moreover, not much is known regarding higher-risk environmental conditions (e.g., walking on a narrow path, stepping over obstacles) in DT setting and the specific features under which older adults shift prioritization. Brustio et al. [27] found a reciprocal influence of motor and cognitive task performances in both younger and older adults. However, they did not compare performances under different task conditions in the older group only. Plummer-D’Amato et al. [28] provided evidence that effects of cognitive task complexity on gait speed of older adults were moderated by the environmental constraints on locomotion. However, they used executive function tests mainly relying on inhibition. While this core executive function jointly contributes with physical fitness to gait performance [7], another core executive function, working memory, seems relevantly associated with gait performance in aging [29]. 

Individual factors such as lifestyle habits may also moderate DT effects at old age. Beneficial effects of physical activity on cognitive functions with advancing age have been reported. However, it is still unclear whether physical activity is beneficial in preventing cognitive and DT performance decline in older individuals [30,31,32]. Cross-sectional and longitudinal evidence supports the notion that physical activity levels predict both gait and cognitive performance at old age [33,34], and that walking habits are associated with better motor–cognitive DT mobility of older individuals [35].

Thus, the first aim of the present study was to evaluate the interactive effects of cognitive complexity and environmental constraints on DT walking performance in older individuals to further our understanding on the strategies of cognitive resource allocation and task prioritization. The novelty of the present aim was the manipulation of the level of executive function involvement in DT along with the manipulation of environmental constraints on locomotion. Executive function involvement was manipulated using two different cognitive tasks: one mainly challenging the working memory span; and the other challenging both the ability to update and monitor working memory contents and to inhibit mental routines [36,37]. Environmental constraints on locomotion were manipulated using flat walking and walking over obstacles [28] in line with the large body of DT research using obstructed walking. Obstructed walking is of interest because of its attention demands and general relevance to community ambulation [38,39]. We expected reciprocal DT effects of the cognitive task on gait performance, with larger costs when coupling higher executive function demands and obstructed walking [38,39]. In older adults, performing a cognitive task strongly relying on executive function should interfere with the cognitive control requirements of stepping over obstacles because of competition for shared resources. 

The second aim of the present study was to investigate whether physical activity levels might attenuate the age-related loss of ability to cope with locomotor tasks. Since more active older adults seem to require less cognitive resources for motor planning and control [40], we expected that higher levels of physical activity could especially counterbalance the difficulty in performing DTs requiring high executive function involvement.

## 2. Materials and Methods 

The study used a cross-sectional design and was conducted in accordance with the Declaration of Helsinki. Following approval by the local university’s ethics committee (Rome, Italy, reference number: Prot. 451/13), recruitment of participants was carried out through flyers and presentation of the study in different senior centers and trade unions. After consent procedures, 135 participants (aged between 55 and 88 years, mean age 68.1 ± 8.4; 64 men and 72 women) were screened for health status using the AAHPERD (American Alliance for Health, Physical Education, Recreation and Dance) exercise/medical history questionnaire [41] (Table 1). 

Participants also completed the 12-item Short Form Health Survey Version 21 (SF-12v2) for functional health and well-being assessment [42]. The scores were aggregated into two summary measures ranging from 0 (i.e., lowest level of health) to 100 (i.e., highest level of health): the Physical Component Summary (PCS) and the Mental Component Summary (MCS). Lastly, participants performed the Trail Making Test, which in the present study, was used as a convenient indicator of eventual executive dysfunction and underlying cerebral degeneration or damage [43,44] that might affect the DT effects of interest. Participant characteristics are reported in Table 1.

### 2.1. Procedures

In the laboratory, participants were asked by a trained evaluator to perform cognitive and gait tests (described below) as single task (ST) and combined in DT [45]. Both ST and DT were simple or complex depending on the cognitive demands and the environmental constraints on walking described in Table 2. The conditions, performed in counterbalanced order to avoid practice effects, lasted up to 2.5 min each. Testing lasted about two hours including familiarization and rest intervals. At the end of each task performance, participants rated their effort by using the Borg rating of perceived exertion (RPE) CR10-scale [46]. This is a category-ratio scale anchored at the number 10, which represents extreme effort. It is a feasible tool for assessing the perceptual responsiveness to effort also at older age [47]. 

#### 2.1.1. Cognitive Assessment in ST and DT

To manipulate the cognitive task demands, the backward counting (BC) and the random number generation task (RNG) were used. Backward counting is the most commonly used secondary task in research on gait-related DT effects in aging [32], whereas the RNG concurrently performed with walking has been seldomly used [48]. The choice of the RNG task and of the BC is justified by their commonalities and differences. Both use digits in a verbal task that can be easily performed while walking. The RNG, however, is more challenging.

Backward counting test: Participants were required to count backward out loud from 50 by 2 s. The task was concluded when the participants reached 0 and the time taken was recorded with a stopwatch. The number of correct calculations, errors, and repetitions were calculated and normalized per second. This test represents a working memory task mainly assessing memory span with low demands on updating, as it requires maintaining the last spoken number in mind until the next number is told; it also limitedly challenges inhibition to avoid counting by one.

Random number generation task: Participants were required to generate random number sequences. Keeping track of recent responses and comparing them to a concept of randomness, which is a central aspect of RNG, requires the updating ability to monitor response distribution while inhibiting stereotyped responses [37]. This task is believed to reflect executive processing functions that depend on the integrity of the prefrontal cortex, in particular working memory updating and inhibition [49,50], and is feasible with older adults [51]. The test required participants to verbally generate a sequence of 100 numbers, chosen randomly between 1 and 9, at a frequency of 40 bpm paced by a metronome. A practice trial always preceded the test. The generated numbers were manually and electronically recorded to elaborate the randomness of the sequence. Six indexes were obtained through RNGcalc software [52], three reflecting working memory updating, three for inhibition (Table 3). Data were z standardized; those with higher values indicating worse performance were reversed (redundancy, coupon, runs, and adjacency) and separate summary indices, one for working memory (mean of redundancy, coupon, and mean repetition gap) and one for inhibition (mean of turning point index, runs and adjacency), were calculated with higher values corresponding to better performance. 

#### 2.1.2. Gait Assessment in ST and DT

Gait assessment and analysis were performed using a photocell system (Optojump Next, Microgate, Bolzano, Italy, 19, Software version 1.9.7.0) consisting of 10 transmitting and 10 receiving optical bars placed parallel to each other at 2 m, for a total length of 10 m, each containing 96 light-emitting diode (LEDs). Participants were asked to walk at habitual speed on a rectangular path of 10 × 2 m, for 2.5 min (optical bars placed on right and left side). To exclude acceleration and deceleration phases from the analysis of the gait parameters, the first and the last bars of the Optojump (the first and the last meter) were not considered. Participants performed the walking task under two environmental conditions: flat and stepping over two obstacles of different height (6 and 30 cm) added both ways at 4 and 6 m, respectively. 

Quantitative gait parameters were calculated by the Optojump system to obtain mean values of speed (m/s) and stride length (m) (mean value of stride length normalized for height) and coefficient of variation (CV: % standard deviation/mean) of speed and stride length. Mean and CV indices of gait speed and stride length and related variability indices were chosen because they are considered good indicators of walking assessment in older people [13]. Especially, gait speed is a parameter of gait performance with clinical relevance [53] most sensitive to motor–cognitive interference effects across a range of tasks [12]. 

#### 2.1.3. Computation of DT Effects for Cognitive and Gait Variables

Dual-task effects on cognitive performance: Since the BC and RNG performance indexes were normalized or z-standardized data, simple DT-ST differences were computed. Two DT effect (DTE) variables were obtained, one for flat walking (DTE_flat_) and one for obstructed walking (DTE_obstacle_), separately for the BC index of working memory span and for the RNG indexes of working memory updating and inhibition. 

Dual-task effects on gait performance: For mean speed and mean stride length, we calculated relative DTE as follows [32]:

DTE = [(DT − ST) / ST] × 100%(1)

For CV speed and CV stride length, for which the higher the value, the worse the performance, DT effects were calculated altering the formula as follows [32]:

DTE = [− (DT − ST) / ST] × 100%(2)

In this way, for all mean and CV gait variables, negative DT effect values indicate deteriorated performance in DT (i.e., DT cost), whereas positive values represent an improvement in DT respective to ST (i.e., DT benefit). Given the two different DT walking conditions with concurrent BC or RNG, two DT effects were computed with reference to ST walking: DTE_BC_ and DTE_RNG_.

#### 2.1.4. Assessment of Covariates 

Physical activity level: Daily steps were monitored as a proxy indicator of participants’ level of habitual physical activity. Measurements of the number of steps performed per day were taken one week prior to the commencement of the study for seven days through an inertial sensor (Sensewear Pro3 Armband—Bodymedia, Pittsburgh, PA, USA), worn by all participants on the upper-posterior region of their right arm, the whole day, except while sleeping. A specific software (Inner View, Bodymedia, Pittsburgh, PA, USA) was used to set the device with information regarding sex, age, weight, height, and dominant arm of the participant, and to provide a final report of the number of detected steps to be used for the statistical analysis. 

Executive functioning: The Trail Making test is a paper-and-pencil test composed of two parts: part A, which is a measure of attention and speed, and part B, which, after accounting for part A, is a measure of executive function and specifically cognitive flexibility [44,54]. After demonstration on a sample sheet, the test was performed. Time was recorded in seconds. As correction of errors forms part of the completion time for the task, any errors were pointed out immediately by reminding the participant of the correct task performance. They were then asked to revert to the last correct point of the test and continue from there. A score was calculated by subtracting time of part A from time at part B (Delta Trail Making).

### 2.2. Statistical Analysis

Data were analyzed using the Statistical Package for the Social Sciences, version 21.0 (SPSS Inc., Chicago Illinois). The level of statistical significance was set at *p* < 0.05 for all computations. To check for collinearity among the gait parameters, bivariate Pearson’s correlations were performed with values of coefficients *r* > 0.80 considered as indicators of collinearity. No collinearities were observed. The cut-offs for effect size values of partial eta squared (*η_p_*^2^) were: 0.01 small, 0.06 medium, and 0.14 large.

To investigate the hypotheses regarding the interactive effects of cognitive demands and environmental constraints on DT walking performance (first aim of the study), a repeated measure 2 × 2 multivariate analysis of covariance MANCOVA and subsequent analysis of covariance ANCOVAs were applied to DTEs computed for gait variables (mean and CV speed and stride length). The within-participants factors were “cognitive task demands” (DTE_BC_ versus DTE_RNG_) and “environmental constraints on walking” (flat versus obstacle). Age, physical activity level, and executive functioning were used as covariates. Repeated measures MANCOVA and subsequent ANCOVAs were also applied to DTEs computed for cognitive variables (working memory span and updating, inhibition), with “environmental constraints on walking” (DT_flat_ versus DT_obstacle_) as the within-participant factor and age, physical activity level, and executive functioning as covariates. Post-hoc analysis was performed with planned *t*-tests with Bonferroni corrections for multiple comparisons. 

To investigate the hypotheses regarding the potential of habitual physical activity to counterbalance the difficulty in performing DTs (second aim of the study), regression analyses were performed. The DTE variables showing, in the previous ANCOVAs, significant interactive effects of cognitive and environmental conditions with physical activity were submitted to separate linear regression analyses with physical activity as predictor. 

## 3. Results

### 3.1. Descriptive Statistics

Descriptive statistics (means and SD) and related DT effects are reported in Table 4 for gait variables and in Table 5 for cognitive variables. Comparing the present values of flat walking speed to those reported by Middleton et al. [53], participants resulted “community ambulator” and on average at the “cross-street safely” threshold. Also, they resulted in the category of those “less likely to be hospitalized” and not “expected to show cognitive decline in the following 5 years”. Moreover, as shown in Table 1, results indicate that participants conformed to the normative data for their age category in the Trail Making Test [55] and in the summary scores of the SF-12 for mental (MCS) and physical (PCS) health perception [56]. Perceived effort was rated as light (mean 1.98, SD 1.06 on Borg’s CR10-scale).

### 3.2. Dual Task Effects on Gait Performance

#### 3.2.1. Effects of Environmental Constraints on Gait

The MANCOVA results showed a main effect for environmental constraints on gait (Wilks *λ* = 0.93, *F*(4,128) = 2.49, *p* = 0.046, *η_p_*^2^ = 0.07) and a significant interaction of environmental constraints with the covariate physical activity (Wilks *λ* = 0.94, *F*(4,128) = 2.60, *p* = 0.039, *η_p_*^2^ = 0.07). Subsequent ANCOVAs performed on individual gait variables showed that the main effect was significant for mean stride length (*F*(1,128) = 4.32, *p* = 0.040, *η_p_*^2^ = 0.03) and marginally significant for mean speed (*p* = 0.052), with a significant interaction with physical activity for both variables (mean stride length *F*(1,128) = 9.95, *p* = 0.002, *η_p_*^2^ = 0.07; mean speed *F*(1,128) = 6.94, *p* = 0.009, *η_p_*^2^ = 0.05). The main effect was due to a higher DT cost (i.e., more pronounced reduction of stride length from ST to DT) in the more complex obstructed walking condition (Table 4). 

#### 3.2.2. Effects of Cognitive Complexity on Gait

The MANCOVA results showed a main effect for cognitive task demands (Wilks λ = 0.90, *F*(4,128) = 3.71, *p* = 0.007, *η_p_*^2^ = 0.10) and a significant interaction of cognitive demands with the covariate executive functioning (Δ Trail, Wilks λ = 0.92, *F*(4,128) = 2.75, *p* = 0.031, *η_p_*^2^ = 0.08). Subsequent ANCOVAs showed that both the main effect (*F*(1,128) = 4.30, *p* = 0.040, *η_p_*^2^ = 0.03) and the interaction with the covariate Δ Trail (*F*(1,128) = 7.07, *p* = 0.009, *η_p_*^2^ = 0.05) were significant for CV speed. The main effect was due to a higher DT cost (i.e., larger increment in gait speed variability from ST to DT) in the DT walking with RNG (Table 4).

#### 3.2.3. Interactive Effects of Environmental Constraints and Cognitive Demands on Gait

**Figure 1:**
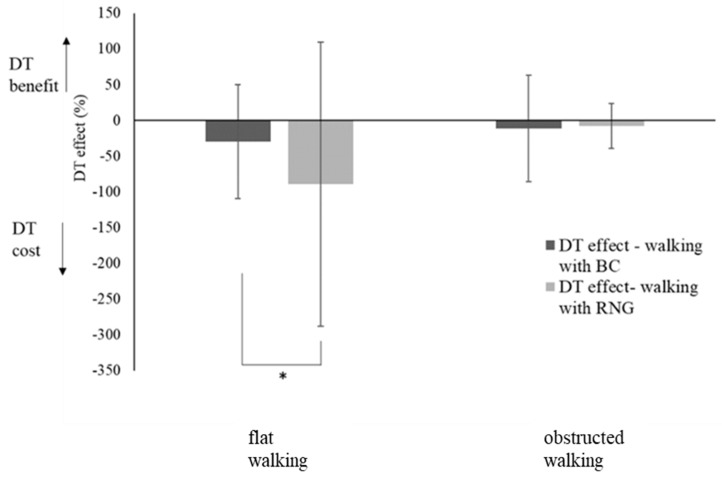
Effect of cognitive task demands on dual task (DT) effects for speed variability (coefficient of variation (CV) speed) as a function of environmental constraints on walking. BC = backward counting; RNG = random number generation. DT effect = ((DT – ST) / ST × 100). * = *p* < 0.05.

The MANCOVA results also showed a significant interaction between environmental constraints and cognitive demands (Wilks *λ* = 0.92, *F*(4,128) = 2.72, *p* = 0.033, *η_p_*^2^ = 0.08), as well as a three-way interaction with the covariate executive functioning (Wilks *λ* = 0.92, *F*(4,128) = 2.73, *p* = 0.032, *η_p_*^2^ = 0.08). Subsequent ANCOVAs showed that these interactions were significant for CV speed (Environmental Constraints × Cognitive Demands: *F*(1,128) = 9.24, *p* = 0.003, *η_p_*^2^ = 0.07; Environmental Constraints × Cognitive Demands × Δ Trail: *F*(1,128) = 10.19, *p* = 0.002, *η_p_*^2^ = 0.07). Post-hoc analysis of the two-way interaction (adjusted *p* = 0.025 for two pairwise comparisons) showed significant differences in DT effects for flat walking (*t*(134) = −3.28, *p* = 0.001), but not for obstructed walking (*p* = 0.454). As Figure 1 shows, participants had a larger DT cost (i.e., larger increment in gait speed variability form ST to DT) when performing DT walking with RNG than with BC, whereas they did not show any DT cost while crossing obstacles. In this obstructed condition, participants exhibited overall higher gait speed variability (Table 4).

### 3.3. Dual-Task Effects on Cognitive Performance

**Figure 2:**
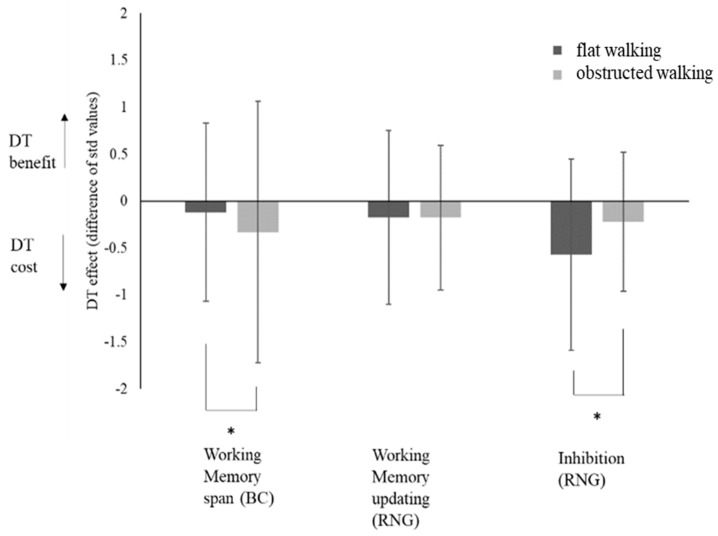
Effect of environmental constraints on walking (flat walking, obstructed walking) on dual-task (DT) effects for working memory span obtained from backward counting (BC), working memory updating, and inhibition obtained from random number generation (RNG). DT effect = ((DT – ST)/ST × 100). * = *p* < 0.05.

The MANCOVA results showed a main effect for environmental constraints on walking (Wilks λ = 0.91, *F*(4,128) = 4.04, *p* = 0.009, *η_p_*^2^ = 0.09) and a significant interaction with the covariate age (Wilks λ = 0.85, *F*(4,128) = 7.83, *p* < 0.001, *η_p_*^2^ = 0.15). Subsequent ANCOVAs performed on individual cognitive variables showed that both the main effect and the interaction with the covariate were significant for inhibition (main effect: *F*(1,131) = 8.40, *p* = 0.004, *η_p_*^2^ = 0.06; interaction: *F*(1,131) = 18.22, *p* < 0.001, *η_p_*^2^ = 0.12) and working memory span (main effect: *F*(1,131) = 3.97, *p* = 0.048, *η_p_* = 0.03; interaction: *F*(1,131) = 5.85, *p* = 0.017, *η_p_*^2^ = 0.04), but not for working memory updating. As Figure 2 shows, there were larger DT costs (i.e., larger decrements in cognitive performance form ST to DT) for working memory span during obstructed walking and for inhibition during flat walking. 

### 3.4. Prediction of Dual-Task Effects by Physical Activity Level

**Figure 3:**
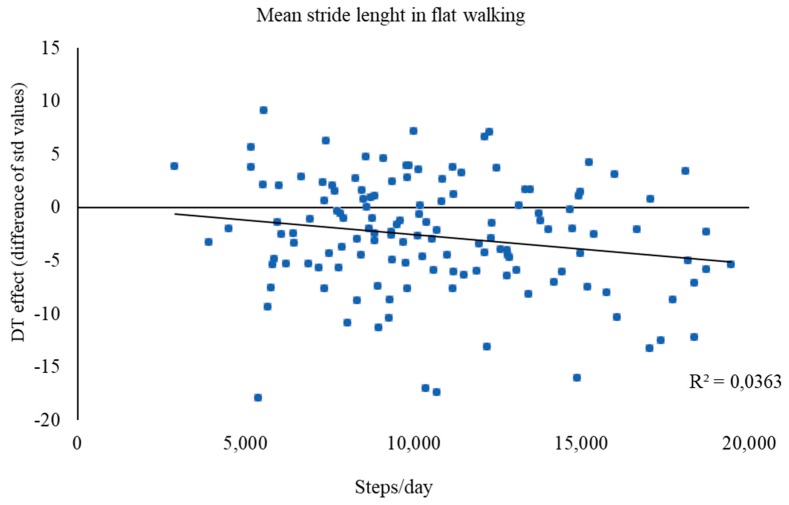
Prediction of dual-task (DT) effects accrued by physical activity level (steps/day) for mean stride length during flat walking.

The MANCOVA/ANCOVA performed on gait data showed interactive effects of physical activity with environmental constraints for DTE for mean stride length and speed. Therefore, these DTE variables (collapsed for dual tasking with BC and RNG) were regressed on physical activity separately for flat and obstructed walking. Physical activity level explained a small but significant percentage of variance of DTE for mean stride length during flat walking only (*R*^2^ = 0.04, *t* = −2.24, *βstd* = −0.19, *p* = 0.027; Figure 3), but not while crossing obstacles. No prediction of DTE for mean speed in either environmental conditions emerged. 

Executive functioning and age, which were included with physical activity as covariates in the MANCOVA/ANCOVAs also exhibited interaction effects with environmental constraints and cognitive demands. Since, however, they did not affect the variables affected by physical activity, they were not entered into subsequent regression analyses as they were not pertinent to the aim of the study. 

## 4. Discussion

The present study investigated the interactive influence of cognitive demands and environmental constraints on DT walking in older individuals. The aim was to frame individual, task, and environmental characteristics as constraints acting on task prioritization [17,19,20,21,22]. Particularly, we evaluated whether adding cognitive tasks with different executive function challenges to walking under flat and obstructed conditions produced reciprocal DT effects that may elucidate task prioritization. Weak to moderate interactive effects of cognitive and environmental conditions were observed, suggesting that older individuals’ prioritization in DT varies depending on the type of the executive functions and environmental constraints. Habitual physical activity weakly affected DT performance in easy environmental conditions (flat walking) only. The spatial characteristics of gait were affected by the environmental demands of DT walking, while the temporal gait characteristics were affected by the cognitive demands of DT walking. 

### 4.1. Reciprocal Dual-Task Effects and Task Prioritization

The most relevant outcome of the study was that in older individuals, the reciprocal influence of concurrent cognitive and locomotor tasks was affected by the type and amount of executive function involvement. A cognitive task performed concurrently to walking affected gait speed, in line with evidence that the effect of cognitive tasks on gait was prominent on speed [12]. Specifically, the locomotor–cognitive dual tasking affected gait speed variability. This gait parameter informs on the ability of the neuromuscular system to regulate gait and maintain stable walking. The higher the variability, the lower the gait stability [57]. 

The DT effect on gait variability differed as a function of the level of environmental constraint on walking. According to our expectation, stepping over obstacles increased demands for resource allocation to gait [27,28]. This is reflected in a generally high gait variability under all ST and DT conditions (Table 4). The difficulty of stepping over obstacles may have introduced a ceiling effect on gait instability that leveled out any differences between ST and DT performances, thus eliminating DT effects (Figure 1, right). The cognitive effort required to negotiate obstacles also determined a withdrawal of resources from the concurrent cognitive task, as reflected in larger DT costs at the expense of working memory span performance (Figure 2, left). The observed decrement in cognitive performance found when stepping over obstacles shows that older individuals prioritize gait performance to cope with the increased requirements of cognitive control for gait regulation. This may reflect an age-related loss in automaticity [10]. 

A different pattern of resource allocation to concurrent cognitive and locomotor tasks was observed when older individuals walked unobstructed. Task prioritization was affected by the different level of executive control and working memory required to perform the cognitive task. When it challenged only working memory span (counting backwards), aging individuals seemed able to find an appropriate trade-off in resources allocation. In fact, they showed smaller DT costs on gait as compared to walking while counting randomly (Figure 1, left). To preserve gait performance from DT costs they seemingly did not withdraw resources from the counting task, as suggested by a negligible DT cost on working memory span performance (Figure 2, left). 

This balance in resource sharing was no longer observable when performing a task that challenged executive control and working memory to a higher extent. In fact, counting randomly worsened walking performance more than the simpler backward counting (Figure 1, left), and elicited a stronger competition for shared resources that led to use of mental routines instead of properly inhibiting them (Figure 2, right; larger DT effect in flat walking). This supports the notion that more difficult cognitive tasks may amplify detrimental DT effects on gait speed even when walking with low environmental constraints [28]. 

The negligible DT effects on memory updating performance (Figure 2, middle) were unexpected, but added nuanced information on which executive function components compete for common resources in motor–cognitive dual tasking. Our results suggest that the control of locomotor movements relies on the capacity of the memory span rather than on the ability to manipulate the information held in working memory. While working memory seems relevantly associated with gait performance in aging [29], there is evidence implicating the role of inhibition on motor–cognitive DT interference [28]. The DT effects observed for the inhibition index of RNG suggest that the inhibition of mental routines is a sensitive indicator of loss of cognitive control and reliance on more automatic mental routines when resources must be allocated to walking. Thus, divergences on the effects of cognitive task demands on DT walking performance [23,24] might be resolved by a nuanced consideration of the interplay between executive function involvement and environmental constraints on walking. However, considering the huge standard deviation of RNG performance (Figure 2), it cannot be excluded that a high interindividual variability in coping with high task demands may have outweighed potential DT effects on working memory updating.

Taken together, reciprocal DT effects of cognition on movement and of movement on cognition suggest that if, as in our study, no instruction is given regarding which performance dimension to prioritize, a variable task prioritization is used probably depending on the perceived difficulty of the concurrent tasks [17]. Older adults seem able to balance resource sharing, limiting reciprocal DT costs, when they perform a low-challenging working memory span task during simple, unobstructed walking. Instead, they seem to prioritize motor performance in more demanding environmental conditions such as obstructed walking, drawing on the limited-capacity resources of their working memory span to control gait. This is consistent with the type of dynamic task prioritization found in young adulthood [58], which becomes particularly meaningful for fall prevention and safety in older adulthood.

### 4.2. Dual-Task Effects on Gait: Influence of Physical Activity

The second aim of the study was to determine whether higher levels of habitual physical activity would offset the detrimental effects of aging on dual-task walking. The hypothesis was that higher physical activity levels would predict lower DT costs. Regression analyses revealed only a weak beneficial effect of higher physical activity levels. Specifically, the more active older adults are, the more they seem able to maintain stride length also when concurrently performing cognitive tasks that dip into capacity-limited resources. This beneficial effect has clinical relevance at old age [53]. Particularly, the significance of gait speed, which declines with age, lies on its link to several health outcomes [59], such as ability/disability [60], maintenance of balance/falling [61], and survival/mortality [62]. The positive association of spatial gait features in DT with daily steps suggests that physical activity habits involving locomotion have a protective effect on the age-related decline in DT mobility that mirror everyday task demands. Indeed, a meta-analysis showed that different types of structured physical activity (resistance, coordination, and multimodal training) all have beneficial effects of large size on gait at old age [63]. 

In contrast, being physically active seems beneficial only to simple, unobstructed walking, since the number of daily steps habitually performed was predictive of lower DT costs only in flat walking (Figure 3) but not in the presence of higher environmental constraints. The absence of association of daily steps with any other gait parameter during more complex DT walking suggests that habitual physical activity is necessary but not sufficient for older adults to maintain an overall DT walking ability. Thus, it is probably necessary for older individuals to engage in specific motor–cognitive DT training beyond simple walking [64].

### 4.3. Limitations

Some limitations of the study must be mentioned. The study is cross-sectional in nature and therefore could not directly address causality. Effect sizes were medium in the MANCOVAs and small in the regressions, possibly due, at least in part, to the high inter-individual variability in gait and cognitive performance. We do not have available reference data to establish the clinical relevance of the findings. The intensity of the locomotor exercise task was neither manipulated nor assessed objectively. While there seem to be small and positive effects of acute exercise bouts, also of light intensity, on concurrent cognitive performance, our walking task duration was below the 10-min threshold for acute exercise to affect cognition [65]. Moreover, while inertial sensors allowed collecting objective data on locomotion-related physical activity levels, they could not provide information on motor activities other than locomotor ones. These, in turn, may have an impact on postural balance and muscle strength, which jointly contribute to walking performance particularly when the gait task has high motor or cognitive requirements [66]. Moreover, the possible ceiling effect on gait performance encountered for the more complex obstructed walking might be overcome in future studies employing an ecological approach to DT performance in aging [67]. Our study was already in line with the ecological approach regarding the use of laboratory tasks that are close to real-life conditions and the investigation of reciprocal DT effects. Nevertheless, we did not use adaptive testing or testing-the-limits approaches for adapting task difficulty and controlling for the influence of practice [17].

## 5. Conclusions

In conclusion, the present study furthers our understanding of how older adults cope with DT walking. The innovative contribution consists in gaining a more fine-tuned appreciation of task prioritization and its individual-level and environmental moderators. The investigation of the first aims suggests that resource allocation policy and task prioritization depend on the interplay between cognitive task demands and environmental constraints on walking. Regarding the second aim, being active at late-middle and old age seems to slightly benefit unobstructed DT walking performance, probably because locomotor skills are preserved to a degree that simple walking tasks are still performed in an automated manner, sparing attentional resources. 

Therefore, our results, though cross-sectional in nature, are in line with the call for walking and walking promotion interventions [68] targeted to increasing daily steps to reach normative thresholds for health [69]. However, functional mobility needed in daily life also encompasses complex forms of DT walking which, in the present study, seemed unaffected by the number of daily steps. Thus, to maintain ability in more complex DT walking, specifically designed training interventions may be needed. Conclusions from reviews of ST and DT training involving static and dynamic (walking) balance suggest that task-specific DT interventions are most beneficial. This is especially the case for interventions with incremental difficulty, appropriate intensity and duration and variable task prioritization [64]. However, these types of interventions are usually designed for indoor training. Future cross-boundary research should embed nuanced DT walking into more ecological natural environments, which may provide emotionally salient experiences [70] and have the potential to restore attentional resources especially crucial in aging to face everyday life challenges [71,72].

## Figures and Tables

**Table 1:** Participant characteristics: gender, anthropometric data, physical activity (number of daily steps), education, number of medications and diseases, retirement, smoking, and alcohol habits, mean values of summary scores of the Short Form Health Survey 12 SF12 for physical (PCS) and mental (MCS) health perception and executive function (Delta Trail Making).

Gender Female (*n*) Male (*n*)	72 (54%) 63 (46%)
Height (cm)	165.0 ± 90.0
Body mass (kg)	72.4 ±12.6
BMI (kg/h^2^)	26.5 ± 3.5
Daily steps (mean ± SD)	10,669 ± 3671
Educational level <High school (*n*) High school (*n*) College (*n*)	35 (26%) 66 (49%) 34 (25%)
Drugs (mean ± SD)	2.8 ± 2.7
Diseases (mean ± SD)	2.4 ± 2.3
Retirement Yes (*n*) No (*n*)	99 (73%) 36 (27%)
Smoking No (*n*) In the past (*n*) Yes (*n*)	69 (51%) 49 (36%) 17 (13%)
Alcohol No (*n*) Occasionally (*n*)	50 (37%) 85 (63%)
SF-12 PCS (pts) MCS (pts)	52.2 ± 7.1 51.5 ± 9.1
Delta Trail Making (s)	49.0 ± 40.6

**Table 2:** Conditions of the factorial experimental design.

	**Environmental Constraints on Walking**		**Cognitive Task Demands**	
Single Tasking	*Low*	*High*	*Low*	*High*
	(1) Flat walking	(2) Obstructed walking	(3) Backward counting	(4) Random number generation
	**Environmental Constraint**		**Cognitive Demands**	
Dual Tasking	*Low–Low*	*Low–High*	*High–Low*	*High–High*
	(5) Flat walking with BC	(6) Flat walking with RNG	(7) Obstructed walking with BC	(8) Obstructed walking with RNG

Note: BC = backward counting; RNG = random number generation.

**Table 3:** Description of the executive function indices obtained from the random number generation test.

Working Memory Updating	
Redundancy	Index reflecting the unbalance of response alternative frequencies in a sequence of generated numbers based on the theoretical frequencies of each digit
Coupon	Index of the mean number of responses given before all the alternative responses are used
Mean Repetition Gap	Index of the average quantity of digits between successive occurrences of the same number calculated for all digits throughout the whole sequence
**Inhibition**	
Turning Point Index	Index of the similarity between the real frequencies of changes between ascending and descending series of numbers and their theoretical frequency in random responses
Runs	Index of variability of the number of digits in successive ascending or descending runs
Adjacency	Index of the relative frequency of pairs of adjacent ascending or descending numbers

**Table 4:** Means and standard deviations (*n* = 135) of gait variables in all testing conditions: low versus high environmental constraints on walking (flat versus obstacle) in single task (ST) or dual task (DT) with low versus high cognitive demands (backward counting (BC) versus random number generation (RNG)).

		Single Task	Dual Task_BC_	Dual Task_RNG_	DTE_BC_ %	DTE_RNG_ %
Flat walking	Mean Speed (m/s)	1.35 ± 0.19	1.38 ± 0.22	1.13 ± 0.28	2.83 ± 12.64	−16.44 ± 16.30
	CV Speed (sd/mean × 100)	3.53 ± 1.20	4.27 ± 2.36	6.19 ± 5.83	29.65 ± 79.40	88.96 ± 198.80
	Mean Stride length (m)	0.85 ± 0.08	0.86 ± 0.08	0.79 ± 0.08	1.69 ± 6.13	−7.07 ± 6.39
	CV Stride length (sd/mean × 100)	2.98 ± 1.44	3.85 ± 2.59	3.69 ± 1.56	39.69 ± 94.82	33.85 ± 58.61
Ostacle walking	Mean Speed (m/s)	1.15 ± 0.16	1.19 ± 0.19	1.04 ± 0.21	3.56 ± 11.13	−9.17 ± 12.31
	CV Speed (sd/mean × 100)	12.59 ± 4.30	13.23 ± 5.31	13.06 ± 4.53	11.42 ± 74.16	7.75 ± 31.31
	Mean Stride length (m)	0.84 ± 0.07	0.85 ± 0.07	0.82 ± 0.08	1.65 ± 5.84	−3.18 ± 5.19
	CV Stride length (sd/mean × 100)	9.60 ± 7.38	8.48 ± 2.72	8.70 ± 2.19	−0.66 ± 43.70	1.02 ± 35.93

Note: DTE = dual task effect; CV = coefficient of variation. Negative DT effect values indicate deteriorated performance in DT (i.e., DT cost), whereas positive values represent an improvement in DT in respective to ST (i.e., DT benefit) for both mean and CV gait parameters.

**Table 5:** Means and standard deviations (*n* = 135) of cognitive variables in all testing conditions: low versus high cognitive demands (working memory span obtained from backward counting (BC) versus working memory updating and inhibition obtained from random number generation (RNG)) in single task (ST) or dual task (DT) with low versus high environmental constraints (flat versuss obstacle).

	Single Task	Dual Task_flat_	Dual Task_obstacle_	DTE_flat_	DTE_obstacle_
Working Memory span (std score)	0.15 ± 1.17	0.03 ± 0.90	−0.18 ± 0.89	−0.12 ± 0.95	−0.33 ± 1.39
Working Memory updating (std score)	0.12 ± 0.74	−0.057 ± 0.91	-0.06 ± 0.70	−0.17 ± 0.93	−0.18 ± 0.77
Inhibition (std score)	0.26 ± 0.51	−0.31 ± 0.99	0.44 ± 0.56	−0.57 ± 1.02	0.22 ± 0.74

Note: DTE = dual task effect.
